# Polyphenols—Gut Microbiota Interrelationship: A Transition to a New Generation of Prebiotics

**DOI:** 10.3390/nu14010137

**Published:** 2021-12-28

**Authors:** Diana Plamada, Dan Cristian Vodnar

**Affiliations:** 1 Faculty of Food Science and Technology, University of Agricultural Sciences and Veterinary Medicine, Calea Mănăștur 3-5, 400372 Cluj-Napoca, Romania; diana.plamada@usamvcluj.ro; 2 Institute of Life Sciences, University of Agricultural Sciences and Veterinary Medicine, Calea Mănăștur 3-5, 400372 Cluj-Napoca, Romania

**Keywords:** polyphenols, prebiotics, gut microbiota, bioactive compounds, bacteria

## Abstract

The present review summarizes the studies carried out on this topic in the last five years. According to the new definitions, among all the compounds included in the group of prebiotics, polyphenols are probably the most important secondary metabolites produced by the plant kingdom. Many of these types of polyphenols have low bioavailability, therefore reaching the colon in unaltered form. Once in the colon, these compounds interact with the intestinal microbes bidirectionally by modulating them and, consequently, releasing metabolites. Despite much research on various metabolites, little is known about the chemistry of the metabolic routes used by different bacteria species. In this context, this review aims to investigate the prebiotic effect of polyphenols in preclinical and clinical studies, highlighting that the consumption of polyphenols leads to an increase in beneficial bacteria, as well as an increase in the production of valuable metabolites. In conclusion, there is much evidence in preclinical studies supporting the prebiotic effect of polyphenols, but further clinical studies are needed to investigate this effect in humans.

## 1. Introduction

Because microbiota plays such an essential role in human health, it has become a well-researched field over the past 20 years. However, there is evidence about the identification of microorganisms, since 1677, when Antonie van Leeuwenhoek described them as “animalcules”. The relevance and significance of comprehending the interrelationship between host and their resident bacteria populations are extremely necessary to improve the quality of life, prevent the risk of disease, or even treat specific pathologies [1,2,3]. Microbiota composition varies depending on the colonization location. Most of the microbiota components are harmless or beneficial in terms of human health. However, some are detrimental to our health, and a disruption of the equilibrium can trigger the development of dysbiosis. This imbalance can affect systems connected to the microbiota, leading to various pathologies. The human microbiome is considered a superorganism through its taxonomically complex and ecologically dynamic nature, with all the implications in human health [4,5].

The human microbiota is composed of bacteria, fungi, archaea, viruses, and protozoans and can be found in many areas of the body; as it colonizes the skin, mouth, vagina, gastro-enteric tube, and/or respiratory system [6]. Over 70% of microbiota colonizes the gastrointestinal tract (GIT) and contains more than 100 trillion microbes [7]. In the gut microbiota, the major phyla are Firmicutes, about 64%, encompassing genera *Lactobacillus*, *Bacillus*, *Clostridium*, *Enterococcus*, *Ruminococcus*, *Eubacterium*, *Faecalibacterium*, and *Roseburia*. The second phylum is Bacteroidetes, about 23%, comprising *Bacteroides* and *Prevotella*, followed in descending order by the phyla *Actinobacteria*, about 3% *Verrucomicrobia* 2% [7,8].

Human microbiota differs from individual to individual and is influenced by several endogenous and exogenous factors that can affect the constitution and stability of the microbiome. For example, endogenous factors such as age, genetics, birth gestational date, mode of delivery at birth, infant feeding method, weaning period, hormonal changes, health status; and exogenous factors such as diet, the use of medications, especially antibiotics, exercise frequency, climate, geographical region, pollution level, and the level of stress management [9,10,11,12,13,14,15,16].

Over the last years, gut microbiota gained more attention, and multiple studies have been performed considering the interaction between the human diet and the intestinal microbiome. All these studies comprise elaborate research, and their applicability to the human microbiota has progressed considerably in terms of food analysis, the interaction between certain bacterial strains, and interaction between specific food compounds [7,17].

The key roles of the gut microbiota are very important and accomplish essential functions for the host. Among its major roles are: maintaining the structural integrity of the endothelial barrier, influencing the growth of the immune system, providing antimicrobial protection, impacting the metabolism of carbohydrates, lipids, and proteins, and playing an important role in the synthesis of several vitamins such as vitamin K, biotin, cobalamin, folates, nicotinic acid, pantothenic acid, pyridoxine, riboflavin, and thiamine [1,7,18,19].

In addition to the studies conducted on the intestinal microbiota, there are two essential branches related to intestinal microbiota characterization: prebiotics and probiotics. In the case of prebiotics, these are defined as a selective substrate used by the microorganisms offering many benefits to the host such as protective effects on the gastrointestinal (GI) system, central nervous system, immune system, and cardiovascular system. On the other hand, probiotics are defined as living microorganisms, which also provide health benefits such as preventing bowel diseases, improving the immune system, and alleviating postmenopausal syndrome [20,21,22,23,24,25,26].

Over 25 years ago, prebiotics were identified as a class of compounds with the ability to modulate the gut microbiota, conferring health advantages to the host. Since then, the definition of prebiotics has changed. For example, in 1995, prebiotics were defined by Gibson et al. [27] as “a nondigestible food ingredient that beneficially affects the host by selectively stimulating the growth and/or activity of one or a limited number of bacteria in the colon, and thus improves host health”, while the actual definition describes prebiotics as “a substrate that is selectively utilized by host microorganisms conferring a health benefit” [20,27,28].

In line with the last definition, a new class of prebiotics that meet the criteria to be categorized as prebiotic substrate (resistance to the host digestion, the capacity of being fermented by intestinal microorganisms, and the stimulation of the intestinal bacteria), are the polyphenols. The polyphenols’ most essential health benefits are associated with their antioxidant and anti-inflammatory properties. Regarding their role as a prebiotic substrate, the advantages of polyphenols are attributed to the ability of the intestinal microbiota to metabolize phenolic compounds [27,29,30,31,32].

Polyphenols are described as plants’ secondary metabolites, usually found in foods such as cereals, fruits, vegetables, wine, coffee, tea, and many other derived foods. One of the most common issues regarding the properties of the polyphenols is related to their bioavailability, a parameter influenced by multiple factors, such as food processing, food interaction, dietary intake, distribution, food content, and environmental factors. The quality and the number of benefits offered by polyphenols to the host health have a significant impact, improving the quality of life through their ability to influence the modulation of the gut microbiota [33,34,35].

This work aimed to review the latest studies that identified and discussed the role of polyphenols as prebiotics in gut microbiota modulation. In addition, we have presented the approaches applied for investigating the relationship between gut microbiota and various human pathologies, especially those connected with the GIT.

## 2. Prebiotics and Polyphenol Classification

**Figure 1:**
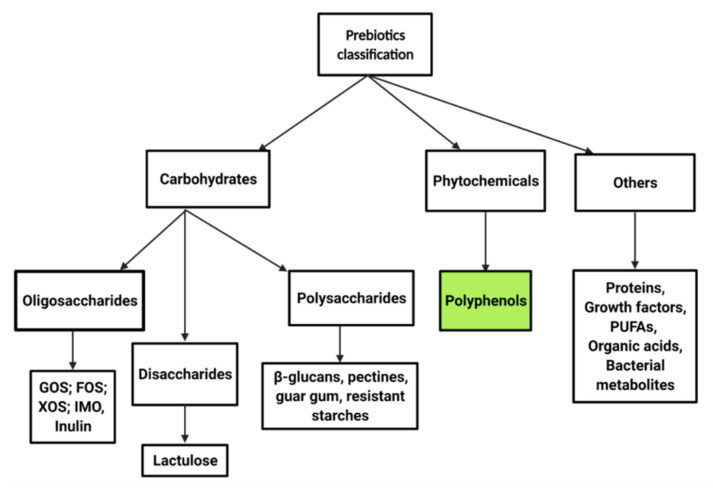
Prebiotic classification (GOS, galactooligosaccharides; FOS, fructooligosaccharides; XOS, xylooligosaccharides, IMO, isomaltooligosaccharides; PUFAs, polyunsaturated fatty acids) [38].

The majority of prebiotics have carbohydrates as a major component, but there are also other classes such as polyphenols, minerals, and polyunsaturated fatty acids that exert the same prebiotic properties [36]. Prebiotics rich in carbohydrates can be found naturally in fruits, vegetables, and foods rich in fructooligosaccharides, xylooligosaccharides, galactooligosaccharides and raffinose oligosaccharides [37]. The following criteria must be accomplished for a food component to be classified as a prebiotic: can resist gastric acidity, hydrolyzation of mammalian enzymes and absorption in the upper GIT, can be fermented by intestinal bacteria, and can stimulate their growth [38]. A straightforward classification of prebiotics is presented in Figure 1.

Polyphenols are a large heterogeneous collection of compounds found naturally in vegetables, fruits, cereals, tea, coffee, dark chocolate, cacao powder, and wine. However, they all have a structural unit in common, hydroxylated aromatic rings or phenolic rings [39]. Considering their composition, polyphenols can be classified into coumarins, phenolic acids, flavonoids, stilbenes, and lignans. In Table 1 the classification of polyphenols along with their subclasses, examples, and food sources are presented [40,41]. The phenolic compounds mentioned in Table 1 are presented as prebiotic substrates. Still, not all of them are absorbed at the small intestine level, and the majority of them reach the colon microbiota serving as substrate for the colonic consortium [33].

## 3. The Concept of Prebiotics

The definition of prebiotics is similar to the definition of dietary fibers. However, the definition of prebiotics is differentiated by the selectivity for certain species. Natural sources of carbohydrate-based prebiotics comprise two main categories: dietary fibers and sugar alcohols. In the case of dietary fibers, these include nondigestible starch polysaccharides (with resistant starch), nonstarch polysaccharides, and sugar alcohols (represented mainly by sorbitol and mannitol), which are derived from simple sugars [54].

Regular fruit and vegetable consumption is part of a healthy diet and brings many benefits through their content of phytochemicals, including polyphenols, polysaccharides, and terpenoids. The main ways in which these phytochemicals bring benefits are through their nonabsorbed fraction that acts as a prebiotic and their absorbed part that induces stress resistance mechanisms. The phytochemical class has a broad range of effects, mainly by modulating gut microbiota and maintaining its homeostasis [55].

By comparison with other compounds, such as carbohydrates, lipids, and proteins, phytochemicals are not required for physiological functions (heart rate, digestive activity), but they can induce biological effects. For instance, after absorption, phytochemicals can improve gut barrier integrity by inducing the expression of tight junction proteins by activating the aryl hydrocarbon receptor in the lumen of the epithelial cells [55].

The evidence of the positive effects of the phytochemicals is constantly growing, especially about polyphenols, as the intake through diet is high, and they play a significant role in gut microbiota modulation. Due to their bioavailability, their effects are closely connected with the food matrix, depending on whether they are consumed as isolated compounds or with the whole food [56]. Moreover, processing conditions such as microencapsulation and nanoencapsulation, enzyme treatment, ultra-homogenization, high hydrostatic pressure, and cold extraction can influence the gut metabolism of polyphenols [57]. Polyphenols do not have a uniform distribution, and they can be found as a mixture, mostly in food products, processed and unprocessed. Their quantity and quality depend on soil type, sun exposure, soil moisture, degree of ripeness at harvest time, processing, storage, and culinary preparation [58].

### 3.1. Polyphenols as a Prebiotic Substrate

Overall, population statistics present an estimated intake of 900–1000 mg/day polyphenols, but this can differ depending on the geographic location and sociodemographics of the targeted group. Among the most frequently consumed sources are: tea, coffee, red wine, fruits, and vegetables. In terms of types of phenolic compounds, procyanidins, flavanols, anthocyanidins, flavonols, and flavanones were among the most frequently identified [58].

The reciprocal interrelation between gut microbiota and polyphenols is a well-known subject of interest, as it can modulate host health. The key factors of this interrelation are the bioactive metabolites. The effect of polyphenols on the intestinal microbiota is achieved by influencing the growth and metabolism of bacteria and by interfering with the cell function of the cell membrane. The majority of the polyphenols can hinder biofilm formation and significant effects via the hindering of bacterial quorum sensing. For example, the flavonol and flavones classes in the Staphylococcus genus can hinder bacterial helicase activities while increasing membrane cytoplasm permeability. Another example is the flavanone-rich citrus extract in combination with the isolated flavanones, as they stimulate a reduction of biofilm formation by inhibiting the quorum-sensing signal mediated by acyl-homoserine lactone. These flavanones can reduce the synthesis of acyl-homoserine lactone and its metabolites [59,60,61].

Polyphenols exert their beneficial effects as prebiotic substrate, on the one hand, by increasing the growth and settlement of the probiotic bacterial families such as *Bifidobacteriaceae* and *Lactobacillaceae* and, on the other hand, by reducing the number of pathogenic bacteria such as *Escherichia coli*, *Clostridium perfringens*, and *Helicobacter pylori* [57,62], a mechanism associated with modification in the permeability and the rigidity of the bacterial membrane through modifications of beneficial and pathological bacteria proportion, a change in the composition of short-chain fatty acids (SCFAs) was observed, together with a decrease in inflammation and obesity incidence [63].

Recent studies demonstrated the beneficial effect of polyphenols by stimulating bacteria such as *Akkermansia muciniphila*, and *F. prausnitzii*, observed in mice trials, that had red grapes in their diet. Furthermore, after 16S rRNA gene sequencing, and quantitative PCR on cecal, and fecal samples, an increase of *A. muciniphilla*, together with a decreased ratio of Firmicutes to Bacteroidetes phylum were observed [64,65]. Moreover, after consuming polyphenols from tea, similar results were observed [66].

The fruit group represents a specific polyphenol group that is widely studied, and the evidence confirms their prebiotic effects. Red grape extract or grape seeds were observed to have many benefits by increasing *Lactobacillus reuteri*, *Lactobacillus acidophilus*, *Clostridiales*, *Ruminococcus*. Berries are another well-studied fruit group that is well correlated with the prebiotic effect of polyphenols, and it was demonstrated that berries can decrease the expression of IL-1β, cyclooxygenase-2, nuclear factor k-light chain enhancer of activated B cell, myeloperoxidase, malondialdehyde, and prostaglandin E2, and increased superoxide dismutase and catalase activities [67,68].

The main sources of polyphenols are carrots, broccoli, beetroot, cauliflower, and potato peel. However, the polyphenol quantity is compared to the fruit group. For example, polyphenols from carrots are much more beneficial to human health when the carrots are consumed entirely, not only as carrot extract. Polyphenols from carrots can increase the growth of *Lactobacillus rhamnosus*, *Bacteroides*, and decrease *Clostridiales*, *Ruminococcus*, *Coprococcus*, *Oscillospira* [69]. In the cruciferous family, polyphenols such as kaempferol, quercetin, sinapic acid, chlorogenic acid, and ferulic acid can be found [70].

Other food groups with an elevated polyphenol content that have a prebiotic effect through interaction with the gut microbes are the beverages, cereals, pulses (lentils, chickpeas, beans, peas, and soybeans), and nuts groups. In the case of the beverages group, there are well-studied compounds such as red wine polyphenols and tea polyphenols. Red wine polyphenols enhanced the concentration of the genera *Prevotella*, *Bacteroides*, *Enterococcus*, and *Bifidobacterium* in several studies on human gut microbiota [71]. After in vitro fermentation, increased levels of *Cloacibacillus*, *Klebsiella*, *Alistipes*, *Akkermansia*, and *Victivallis*, and decreased levels of *Blautia coccoides*, *Bacteroidetes*, *Subdoligranulum*, *Anaeroglobus*, and *Bifidobacterium* were also observed [71]. In regard to the polyphenols from tea, green tea and black tea are among the most studied, with 3-O-methylgallate, gallocatechin gallate, epigallocatechin gallate, epigallotechin, theaflavins, theasinensins, and thearubigins [72] as the most predominant phenolic compounds with prebiotic potential [73,74].

For the cereal polyphenol group, compounds such as hydroxycinnamates acid, ferulic acid, coumaric acid were identified. These compounds have the property to enhance the growth of *Bidifobacteria*, such as *F. prausnitzii*, *Lactobacillus* sp., *A. muciniphila* [57]. The pulses group contains polyphenols as a major antioxidant compound [74]. The most frequently identified are flavones flavanones, proanthocyanidins, tannins, catechins, cyanidin, delphinidin, hydroxybenzoic, and hydroxycinnamic acid, stilbenes, ferulic and sinapic acids. Polyphenols from pulses have the antioxidant capability to inhibit reactive oxygen species. The highest quantity of polyphenols has been found in beans and compounds such as hydroxycinnamic acids, hydroxybenzoic acids, ferulic acids, rutin have been found to enhance the growth of *L. acidophilus*, *L. casei*, *L. delbrueckii*, and to decrease the abundance of *C. perfringens* and *Ruminococcus gnavus* [75]. The nuts group contains as major polyphenol compounds ellagitannins in chestnuts and walnuts, and proanthocyanidins in pistachios, almonds, hazelnuts, and pecans. Primary metabolites include valerolactones, hydroxybenzoic acid, hydroxycinnamic acid, hydroxyphenyl acetic acid, hydroxyphenyl valeric acid, and hydroxyphenyl propionic acid are produced after being metabolized [76,77].

### 3.2. Types of Polyphenols Found in Food and Their Effects on Host Health

**Figure 2:**
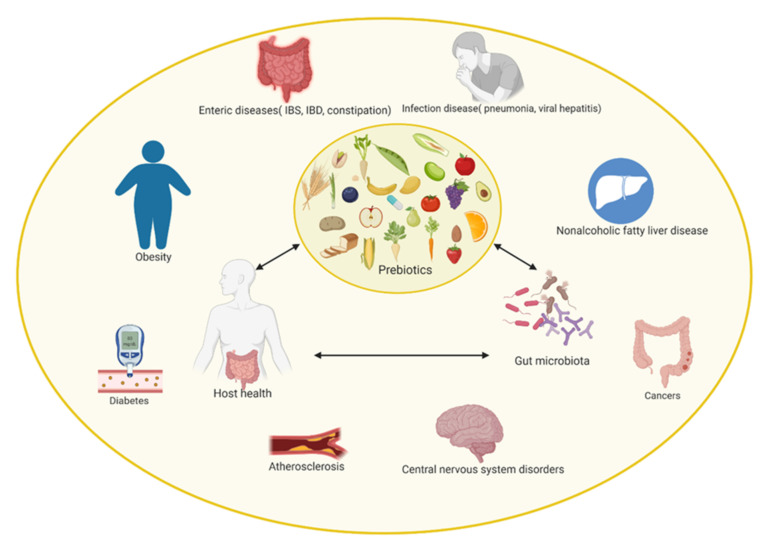
Roles of polyphenols on gut microbiota and implications in human health.

It is well known that polyphenols have beneficial effects on host gut microbiota, and on host health implicitly, as it can be observed in Figure 2. Their role is mainly exerted through their metabolites, with or without other present compounds, such as fiber, and their ability to modify gut microbiota composition. The quantity of dietary polyphenols present in the human diet is remarkable, the main difficulty encountered being the polyphenols’ bioavailability after their bioconversion by the gut microbiota [78].

Hydroxycinnamic acids represent a major contribution from total polyphenol consumption and have effects on cardiovascular diseases, metabolic syndrome, and colorectal cancer [79,80,81]. Gallic acid is another polyphenolic compound present in fruits, vegetables, and herbals that have beneficial effects on human health, such as antioxidant, anticancer, anti-inflammatory, antimicrobial effects. Recently, its role in enhancing gut microbiome activities has been demonstrated [82]. Fruits, vegetables, and beverages (tea, red wine) represent major sources of polyphenols, especially flavonol types. There are three main representative compounds in this category, namely quercetin, myricetin, and kaempferol. These bioactive compounds play an essential role in cardiovascular diseases, have anti-inflammatory activity, and are also involved in gut microbiota modulation. The gut microbiota can transform them, and at the same time, they can influence the composition of gut microbiota [83,84,85,86]. Among flavanones, the most abundant and representative compounds are naringenin and hesperidin, which have anti-inflammatory, antioxidant, and anticancer properties, but they also impact gut microbiota by affecting their bioavailability [87,88]. Among the richest in flavanols, foods that are cocoa-based products, which exert many beneficial effects on blood pressure, insulin resistance, vascular damage, and oxidative stress. New studies present their neuromodulatory and neuroprotective actions in humans, but further research on this topic is needed [89,90,91].

From all plant estrogens, isoflavones have been studied the most, and more precisely, isoflavones from soy and soy products. Other isoflavone sources are green beans, mung beans, and red clover. Isoflavones comprise as main compounds genistein, daidzein, glycitein, and have many beneficial effects in several cancer types, such as breast, and prostate cancer, cardiovascular diseases, osteoporosis, menopausal symptoms, and bone loss [49,92,93].

Initially known for their coloring properties, anthocyanins are a group of polyphenols with health benefits, such as antioxidant and anti-inflammatory actions, reducing the risk of cardiovascular disease, type 2 diabetes, improve weight management, neuroprotection, and can stimulate the growth of *Bifidobacterium* spp. and *Lactobacillus-Enterococcus* spp [94,95,96,97,98]. Similar to apigenin and luteolin, flavones are found in plants such as parsley, celery, onions, fruits, herbs, and plant-based beverages. Studies have found that apigenin and luteolin can inhibit antigen-triggered proliferative responses by autoreactive T-cells, and strongly inhibit the secretion of immune cytokine interferon-gamma (IFN-γ). Apigenin also has positive effects on rheumatoid arthritis, diabetes mellitus, lupus, multiple sclerosis, myocarditis, ulcerative colitis, amnesia, and Alzheimer’s disease [99,100,101,102]. Stilbenes are among the polyphenols largely found in plants and foods such as red grapes, red wine, tea berries, and peanuts, with the ability to enhance the growth of lactobacilli, bifidobacteria, and especially *F. prasnitzii*, which is a butyrate producer. It has been shown that the stilbenes class has antioxidant, anti-inflammatory, antitumor, antiplatelet aggregation, cardio-protective, aging delay effects, and enhances a higher gut microbiome diversity [103,104,105,106]. Lignans are a class of phenolic compounds that can be metabolized to the biologically active enterolignans, enterodiol, and enterolactone by intestinal bacteria. The main sources of lignans are flaxseed, pumpkin seed, sesame seed, soybean, lentils, broccoli, and some berries, the representative compound of this class being secoisolariciresinol. Their positive effects are highly correlated with menstruation and conditions typically associated with menopause and a reduction of the growth of cancerous tumors, especially breast, endometrium, and prostate [107,108,109,110]. Tannins are found in leaves, seeds, barks, roots, fruits, vegetables, legumes, cereals, shrubs, and in more than 40 herbs. They have health effects such as antimicrobial effects, antioxidant, anticancerous, anti-allergic, anti-inflammatory, antihelminthic. In addition, they have effects against *Penicillium* spp., HIV, *S. aureus*, *C. botulinum*, and hydrolyzable tannins have bacterial activity against *H*. *pylori*, by lowering their viability.

On the other hand, nonabsorbable tannins can reach the colonic microbiota, exerting a prebiotic effect, modulating gut microbiota composition and function, and promoting beneficial bacteria’s growth [111,112,113]. Cinnamon is assumed to be the most important source of coumarins, which can also be found in blackberries, cranberries, raspberries, strawberries, cherries, grapes, apricots, sweet clover, woodruff, vanilla grass, and in foodstuffs such as olive oil, soy oil, peanuts oil, corn oil, coffee, nuts, wine, tea, propolis and propolis products. Coumarins have pharmacological applications exerting antioxidant, anti-inflammatory, antitumoral, antimicrobial, antiviral, and neuroprotective activity. In some derivates of coumarins, ammoresinol and ostruthin, have been observed to have significant effects against Gram-positive bacteria, *Micrococcus luteus*, *Micrococcus lysodeikticus*, *S. aureus*, and *Bacillus megaterium* [114,115,116,117].

## 4. Prebiotics as a Nutritional Substrate for Human Gut Microbiota

Many compounds are produced after the bacterial metabolism of macronutrients and micronutrients in the gut, and the most studied are SCFAs after fermentation of dietary fiber. About 5–10% or more of the diet is made of dietary fiber, a nutrient that includes polysaccharides, oligosaccharides, and resistant starch. In the small intestine, they are degraded by the host enzymes. After they pass to the distal gut, they serve as substrates for microbial carbohydrate-active enzymes [118].

**Figure 3:**
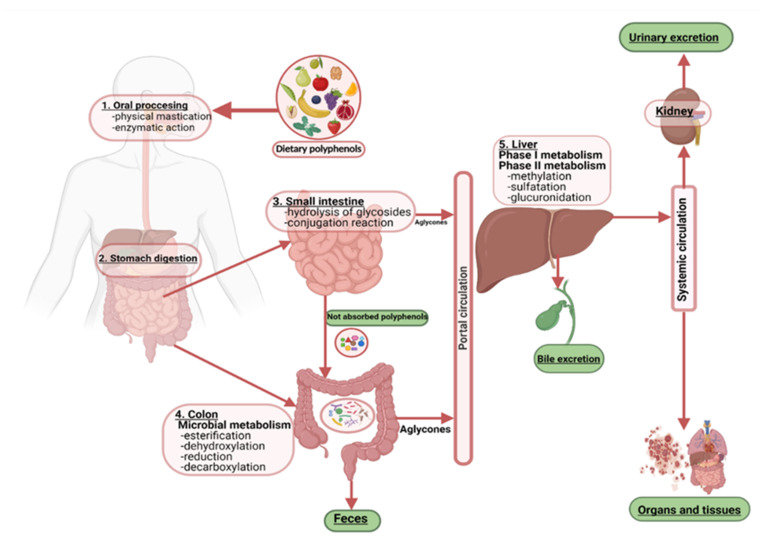
Metabolism of polyphenols by human gut microbiota.

Bioactive compounds such as polyphenols can improve both gut health and overall health status, but the gut microbiota must be abundant and diverse for this to happen. Polyphenols are found as glycosides and complex oligomeric structures in plant foods. These intricate structures are metabolized sequentially in the human body (Figure 3). For example, after the polyphenols reach the distal part of the intestine, they are hydrolyzed and metabolized by the intestinal enzymes and the gut microbiota. Next, the converted polyphenols reach the liver through the portal circulation, where they are subjected to two phases of metabolism, resulting in different metabolic compounds. The next stage is absorption, where they enter phase II metabolism by glucuronyl transferases, sulfate transferases, and catechol methyl transferases, yielding sulfate, glucuronide, and methyl conjugates can be found in the circulatory system. After that, they can be detected in urine three or four days after intake.

Phenolic compounds are generally found conjugated to glycosides, glucuronides, and organic acids, which can be hydrolyzed by gut microbiota, resulting in aglycones. Consequently, after absorption from the colon, follows transformation into phase II conjugates (sulfated and glucuronidated conjugates) in the intestinal tissues and the liver. Conjugated compounds are excreted into the gut as biliary constituents via enterohepatic recirculation, and before being reabsorbed or transformed, microbial enzymes deconjugate these compounds. Fecal microbial enzymes, β-glucosidase, α-rhamnosidase, esterase, β-gluronidase, catalyze the deconjugation in the gut. Reactions such as ring and lactone fission, dehydroxylation, reduction, decarboxylation, demethylation, isomerization are also reactions induced by the gut microbiota. Microbial transformations are influenced by phenolic structure, flavonoid and nonflavonoid factors, polymerization degree, and spatial configuration [119,120].

One of the most common polyphenol groups is the flavonoid group. Flavonols, flavanones, flavan-3-ols, isoflavones, and anthocyanins are all members of this category. They all have the same structure: two benzene rings (ring A and B), linked by a heterocyclic pyrone C-ring. In foods, they are found as glycosides, O-glycosides, and C-glycosides, flavan-3-ols, which are not conjugated. Flavan-3-ols can form either proanthocyanidins or condensed tannins as a whole, and either procyanidins, prodelphinidins, or propelargonidins, when they are solely made up of one compound, epicatechin, epigallocatechin, epiazfelechin. Simple phenolics derived from the A and B rings are released after the gut microbiota broke down the C-ring in different positions. The resulting type of phenolic compounds is affected by the hydroxylation pattern and the position of the B-ring. Thus, in phenolic compounds as flavonols, flavan-3-ols, proanthocyanidins, rendering hydroxyphenyl-propionic acids and hydroxyphenyl acid, the C-ring cleavages are produced at 1–2 and 4–10 bonds or 1–2 and 3–4 bonds. In flavanones and isoflavone groups, the resulting metabolites show that C-ring cleavage is produced at either position 1 and 2, or 4 and 10. The following steps of flavonoid metabolism carried out by gut microbiota are: demethylation and dehydroxylation reactions. The majority of the resulting metabolites are acid of aldehyde phenolics with one, two, or three hydroxyl and methyl ester radicals, for example, 3-(3,4-dihydroxyphenyl)-propionic acid from the flavonol quercetin and equol from the isoflavone daidzein [121,122].

The other group of phenolic compounds is the nonflavonoid group, which, compared to the flavonoid group, has a higher heterogeneity in structure and a higher polymerization level. Compounds such as benzoic acids, hydroxycinnamates, and stilbenes represent this group. Depending on their chemical complexity, they are absorbed to a greater or lesser extent in the small intestine [123]. The most complex phenolics are hydrolyzable tannins, including gallotannins and ellagitannins. These two compounds have monomeric units, gallotannins have monomeric units of gallic acid, and ellagitannins are composed of monomeric units of ellagic acid [124]. More research is needed to describe the deconjugation of several of the ellagitannins. However, the current hypothesis is that the same principle as the one of releasing aglycone from the degradation of the flavone C-glycosides by gut microbiota could be applied [125]. For tannin-O-glycosides, the gut microbiota metabolizes them extensively the same as punicalagin and pedunculagin.

Furthermore, the gut microbiota also transforms gallic acid and ellagic acid [126]. Gallic acid is suitable for decarboxylation and dihydroxylation reactions, while the ellagic acid is suitable for dehydroxylation. Following the dihydroxylation of ellagic acid, the nasutin metabolites are formed. These compounds have two hydroxyl units removed. After ellagic acid is transformed by lactone ring cleavage, decarboxylation, and dehydroxylation reactions, it forms metabolites named urolithins [127]. After lactonases open one of the lactone rings, luteic acid is produced, which is then converted by decarboxylases in the gut to produce pentahydroxy-urolithin, a major role in the production of different urolithins. Further, after dehydroxylations of pentahydroxy-urolithin, tetrahydroxy-urolithins, and trihydroxy-urolithins are produced, compounds that lead to the principal metabolites are detected in vivo, dihydroxy-urolithins urolithin-A (Uro-A), isourolithin-A (IsoUro-A), and the hydroxyurolothin (Uro-B) [128,129,130].

Gut microbiota can transform dietary lignans and produce mammalian phytoestrogens, such as enterodiol and enterolactone. The gut microbiota can act on lignans through reductions, demethylation, dehydroxylation, and lactonization reactions. A complex pathway with several precursors, metabolites, diverse conjugation patterns, and different bacteria species is required to obtain the final product, enterolactone, from lignans [131,132].

Another class of nonflavonoid phenolics, respectively stilbenes, based on a C6-C2-C6 polyphenolic structure, has as a main compound studied, namely trans-resveratrol. The gut microbiota can transform resveratrol O-glucosides such as trans-piceid into resveratrol aglycone through deglycosylation. After absorption, piceid and resveratrol are conjugated in the form of sulfate and glucuronide derivates of the primary circulating metabolites. The first bacterial trans-resveratrol metabolite is dihydro-resveratrol, followed by 3,4′-dihydroxy-trans-stilbene and 3.4′dihydroxydihydro-stilbene [133,134].

One of the simplest groups of nonflavonoid compounds are hydroxycinnamates, including p-coumaric acid, caffeic acid, ferulic acid, sinapic acid, and their esters with quinic, malic, and tartaric acid. All of these compounds are nutrient substrates for the gut microbiota. Hydroxybenzenes (e.g., catechol) are, after decarboxylation, the final hydroxycinnamate metabolites [135,136].

The hydroxybenzoic acids, which are predominantly found in fruits, are the most prevalent microbial metabolites obtained in the gut from phenolic compounds, both flavonoids and nonflavonoids. Microbial decarboxylase enzymes convert phenolic compounds, including gallic acid, protocatechuic acid, and vanillic acid to pyrogallol, catechol, and O-methylcatechol when a free hydroxyl group is present at the 4-position. These metabolites can be further conjugated with glycine with an increased urinary concentration of hippuric acid, but most of them are rapidly absorbed in the GIT [120].

## 5. In Vitro Modulation of Gut Microbiota through Polyphenol Consumption

In vitro studies on polyphenols have demonstrated through extraction, digestion, fermentation, and other types of chemical and microbiological methodologies that they can influence the resident bacteria, increasing or decreasing their population. In addition, food or food groups that are abundant in polyphenols have been analyzed in vitro in order to check their capacity to influence resident microbiota, with or without their other leading roles, as antioxidants and anti-inflammatories. Detailed information on the in vitro studies published so far can be found in Table 2.

Several common phenolic compounds have been observed following the studies conducted on berries. After their extraction and chemical characterization through High Performance Liquid Chromatography (HPLC), compounds such as anthocyanins, flavonols, caffeic acid derivates, ellagic acid derivates, or ellagitannins were identified. A study done in 2020 by Baenas et al. [137] analyzed polyphenols from raspberry by an in vitro fermentation model and metabolites, such as SCFAs. The results showed that the identified polyphenols were mainly hydrolyzable polyphenols found in the insoluble fraction of fibers. They were the primary compounds responsible for the raspberries’ prebiotic effect. This study concluded that, through their antimicrobial and antioxidant effects, raspberry or raspberry extract could be utilized as a prebiotic substrate in foods, functional foods, as well as in dietary supplements [137]. In another review on berries’ polyphenols and their impact on gut microbiota, it was found that a high quantity of polyphenols can reach the colon and so can further produce metabolites. Berries’ polyphenols can produce changes in the bacteria population, enhancing the growth of *Bifidobacterium*, *Lactobacillus*, *Akkermansia*, *Bacteroides*, and *Eubacterium*, and decreasing the number of *Pseudomonas*, *Salmonella*, *Staphylococcus*, or *Bacillus*. The mechanism of in vitro studies still needs more understanding, but their high production of SCFAs has been identified in many studies and it can offer a direction for the prebiotic-like effect of polyphenols [67].

Another type of food with high quantities of polyphenols are grapes. They can be found mainly in the fruit as such, but also in wine or wine by-products, such as grape pomace. The most common polyphenols identified in grapes are quercetin, anthocyanins, anthocyanosides, anthocyanidins, catechins, and proantocyanidins. Previous studies conducted on the fruit, wine, wine industry by-products, and grape extracts have demonstrated the ability of polyphenols to influence the intestinal bacteria, with significant stimulation of the genera *Lactobacillus* and *Bifidobacterium*. Polyphenols were able to be used as a carbon source by these beneficial bacteria. Another review paper, done in 2018 and based on human clinical trials, tested grapes and red wine polyphenols, which showed modifications in the bacteria ratio from gut microbiota. There was an increase of *Enterococcus*, *Prevotella*, *Bacteroides*, *Bifidobacterium*, *Bacteroides uniformis*, *Eggerthella lenta*, *Blautia coccoides*-*Eubacterium rectale* groups, as well as a decrease of *Actinobacteria*, *Clostridium* spp., C. *histolyticum* group. In the case of dealcoholized wine intake, an increase in the *Fusobacteria*, *Firmicutes* population was noted, and a decrease in the *Actinobacteria* population [64,138,139,140].

Mango peel is another high-polyphenol food with a prebiotic effect. The main polyphenols are gallates, gallotannins, flavonoids, ellagic acid, gallic acid, mangiferin, and muclurin derivates. In vitro digestion and fermentation were performed on mango peel, and it was demonstrated that it has a high quantity of indigestible fiber that can be fermented, resulting in a considerable quantity of SCFAs. Additionally, it could enhance the growth of *Bifidobacterium*, *Lactobacillus*, *Dorea*, and *Lactococcus*. In 2019, research evaluating the possible absorption of polyphenols and the antioxidant capacity of a mango by-product snack showed that a snack containing mango peel and mango paste provided a higher amount of polyphenols compared to mango as such [141,142,143].

Tea is one of the most popular beverages and is high in polyphenols. Tea has many varieties, such as green, black, and oolong, with the main polyphenolic compounds being catechins, theaflavins, and thearubigins. In 2018, Sun et al. [144], described an in vitro study conducted on green, black, and oolong tea. They observed the teas’ effects on the gut bacteria. Tea enhances the growth of *Bifidobacterium* and *Lactobacillus*-*Enterococcus* spp., and at the same time increases the production of SCFAs and inhibits the proliferation of *Bacteroides* and *C. histolyticum* groups. In 2020, Xu et al. [145], also demonstrated the beneficial effect of polyphenols from green tea in an in vitro human colon model, through a decrease in the *Firmicutes*/*Bacteroides* ratio, and in 2013, Kerperman et al. [146], showed that black tea stimulates the growth of *Klebsiella*, *Enterococcus*, and *Akkermansia*, while inhibiting the growth of *Bifidobacteria*, *B. coccoides*, *Anaeroglobus* and *Victivallis* [66,144,145,146,147].

Another food with a high prebiotic-like effect that can offer many health benefits is pomegranate or different parts of it, such as the pulp, peel extract, or juice. Previou*s* in vitro studies, including digestion and fermentation, have demonstrated the polyphenols’ ability to enhance the growth of *Enterobacteriaceae*, *Bacteroides fragilis* group, *Clostridia*, *Bifidobacteria*, and lactobacilli. Catechol, gallic acid, coumaric acid, and protocatechuic acid have been identified as the main metabolites of pomegranate urolithins. The stability of phenolic compounds during in vitro digestion was also observed. This could be associated with the food matrix, not only with the initial phenolic composition [148,149,150].

Among polyphenol-rich foods, pineapple is another good candidate. In 2021, an in vitro study done on UV–C irradiated pineapple snack bars, performed by Del juncal-Guzman et al. [151], there were 26 phenolic compounds identified, such as phenolic acids, flavonoids, and hydroxycinnamic acids. UV-C irradiation treatment did not affect the GI content or the release of this type of compound. The nondigestible part was further submitted to fermentation. The fraction was rich in flavonoids such as gallocatechin, which was biotransformed by the gut microbiota to 3-hydroxybenzoic acid, and 4-hydroxyphenyl acetic acid. In another study, also related to pineapple compounds, performed on flours obtained from the pineapple stems and peels, a high content of phenolic compounds was identified [151,152].

## 6. In Vivo Modulation of Gut Microbiota through Polyphenol Consumption

Human gut microbiota has a very complex structure. Due to the fact that clinical trials are not conducted on a number of subjects, it is hard to demonstrate the exact effect of polyphenols in the human body. Therefore, the majority of the viable data translated to humans are obtained from studies performed on animals, usually mice (see Table 3). In 2019, Jiao et al. [154], tested the potential effect of blueberry polyphenol extract on gut microbiota modulation in mice fed with a high-fat diet. The modifications of the gut microbiota and modulation of bacteria were observed after the 16S rRNA gene sequencing of the fecal microbiome. In addition to these changes in bacteria, other important changes have been reported at the body weight gain suppression level, reduction of the total serum cholesterol and triglyceride levels and controlled the lipid metabolism-associated genes. In another study, done in 2021 by Xian et al. [155], the effect of polyphenols from different parts of red raspberry on the gut microbiota of C57BL/6 mice with diet-induced obesity was tested. They identified that the polyphenol extracts from either whole fruit or pulp had effects in mice with obesity. However, the effect from seeds was different, as it only increased *Bifidobacterium* compared to a high-fat diet [154,155,156,157].

Another type of fruit rich in polyphenols is cherries. As mentioned before, they are concentrated in anthocyanins, flavonoids, chlorogenic acid, and neochlorogenic acid. In 2017, Mayta-Apaza et al. [156], identified in a 5-day human dietary intervention study with the administration of tart cherries that there was a response in high *Bacteroides* individuals with a reduction in *Bifidobacterium* and an increase in *Lachnospiraceae*, *Ruminococcus*, and *Collinsella*. On the other side, in low-*Bacteroides* individuals they responded with a decrease in *Lachnospiraceae*, *Ruminococcus*, and *Collinsella*, and an increase in *Bacteroides*, *Prevotella*, and *Bifidobacterium* [157].

Grapes are a rich source of polyphenols, and the wine industry is a good resource for extracting them, using mainly the pulp of the grapes. Moreover, the wine industry generates impressive quantities of grape pomace, a valuable source of bioactive compounds such as polyphenols. Ten subjects with metabolic syndrome and ten healthy subjects were included in a clinical study. Red wine was administered to both groups. A significant increase in fecal bifidobacteria, *Lactobacillus*, and butyrate-producing bacteria, and a decrease in *E. coli* and *Enterobacter cloacae* were observed in metabolic syndrome subjects. This effect resulted consequently in the reduction of metabolic syndrome risk markers. In another comparative study where similar changes were identified, the results supporting the prebiotic role of polyphenols were obtained from nine participants in a clinical study. Red wine, dealcoholized red wine, and gin were administrated for 20 days, and the first two interventions led to an increase of *Bifidobacterium*, *Enterococcus*, and *Eggerthella lenta*. *Bifidobacteria* growth was linked to an increase in metabolites derived from wine anthocyanins. Following gin administration, there were no observed modifications [158,159,160]. Two reviews [160,161], done on the interaction between wine consumption and gut microbiota, have pointed out the beneficial role on gut modulation, but with the specification that there is more research needed in order to clarify the correlation with human health. Both reviews have analyzed this interaction through in vitro studies, animal studies, and human studies. The authors found scientific evidence about the way gut microbiota can be modulated by polyphenols, and as a result, the number of beneficial bacteria increases while the number of pathogenic bacteria decreases. However, as previously mentioned, there is still a need for additional research before being able to have a clear and comprehensive conclusion [160,161].

A fruit rich in polyphenols and recently studied for its profile of bioactive compounds is *Myrciaria jaboticaba* or jabuticaba. The main compounds found are Castalagin, vescalagin, procyanidin A, and ellagic acid. In 2021, Fidelis et al. [162] studied the lyophilized jabuticaba seed extract administrated in yogurt to Wistar rats with cancer. It was observed that in rats with colon cancer that had been given jabuticaba yogurt, the diversity of *Firmicutes*, *Bacteroidetes* and *Proteobacteria* increased. In another study done in 2021, by Trindade et al. [163], in diet-induced obese mice, the administration of polyphenol-rich jabuticaba peel and seed powder contributed to a decrease in weight gain fat accumulation, concomitant with an ameliorated proinflammatory response [162,163].

Tea polyphenols represent one of the most important groups containing bioactive compounds, which can influence the gut microbiota. Whether it is green tea or herbal tea, both can increase and decrease bacteria. A study done in 2015 [149], analyzed the saponins from herbal tea that can influence the mouse gut microbiota. Saponins of ginseng, red ginseng, notoginseng, and *Gynostemma pentaphyllum* were administrated. After the administration, an increase of *Bacteroides*, *Lactobacillus*, and *Bifidobacterium* was observed in the treatment group. Additionally, a significant increase in *Bacteroidetes*/*Firmicutes* ratio following the consumption of *Gynostemma pentaphyllum* and notoginseng was observed. The consumption of Gynostemma improved the growth of *F. prasnitzii* as well. Green tea polyphenols also have an important impact on gut microbiota. Ma et al. [164], analyzed the association between polyphenols from green tea and the maintenance of intestinal redox state. The genera of *Lachnospiraceae*, *Bacteroides*, *Alistipes*, and *Faecalibacalum* were identified as the biomarkers for intestinal redox state, revealing a beneficial impact of tea polyphenols [149,164,165].

The last two types of polyphenols described in Table 3 are *Cyclocarya paliurus* and the polyphenols bound to dietary fiber, specifically carrot dietary fiber. Song et al., in 2020, [166] described the modulatory effect of *Cyclocarya paliurus* flavonoids in the gut microbiota and on liver clock genes of a circadian rhythm disorder, in a study conducted in mice. It was shown that these types of flavonoids can improve the imbalance of microbial structure in the gut caused by circadian rhythm disruption, and also *Firmicutes*/*Bacteroidetes* ratio was significantly decreased in the intervention group. The last described type of polyphenols is the one analyzed in a bound form with dietary fiber, in this case, carrot dietary fiber. In 2020, an in vitro and in vivo study done on polyphenols bound to dietary fiber highlighted that they may contribute significantly to dietary fiber’s fermentation and antioxidant properties through several actions, such as improving gut structure and balance and producing SCFAs [167,168].

Besides the aforementioned polyphenolic groups, there are also isothiocyanates (for example, sulforaphanes and iberin)—the most abundant polyphenolic compounds found in cruciferous plants [169]. It was demonstrated that these bioactive compounds exert suppression and prevent the development of particular pathologies, such as bladder cancer and ulcerative colitis [170,171,172,173]. A study done in 2021, by He et al. [170], using an ex vivo fecal incubation model, has highlighted the role of gut microbiota composition. They proved that microbiota composition could influence the conversion of glucosinolates to nitriles, measured through their chromatographic peak areas [170].

## 7. Conclusions

This review concludes that, through recent studies and their integration in the category of prebiotics since 2016, polyphenols can be used to modulate the intestinal microbiota, a role added to their main properties, which are antioxidant and anti-inflammatory. Numerous studies, both in vitro and in vivo, show the interrelationship between polyphenols and gut microbiota. These compounds, not only by their structure but also by the resulting metabolites, are a substrate for probiotics, resulting in the growth of beneficial bacteria and the reduction of pathogenic bacteria, thereby maintaining host intestinal homeostasis.

However, it is necessary to develop more studies focusing on the polyphenols’ effects in clinical trials, specifically on their metabolic pathways, the evidence observed mainly in the animal in vitro studies. Using advanced techniques such as omics technologies: metabolomics, genomics, metagenomics, trans-genomics, or proteomics; a better understanding of the polyphenols’ action in the living organisms and their consequent metabolites could be provided. Additionally, their prebiotic effect could be clearly defined and used therapeutically.

In conclusion, by summarizing the latest studies that highlight the prebiotic role of the polyphenols, they have the ability to influence both the modulation of the gut microbiota and the host’s general health through their beneficial effects deriving from different mechanisms and impacting multiple organs and systems.

## Figures and Tables

**Table 1:** The classification of polyphenols was adapted after Wiciński et al. [40].

Class	Subclass	Examples of Compounds	Source	References
Coumarin	Simple coumarins Furanocoumarins Dihydrofuranocoumarins Pyranocoumarins Phenylcoumariuns Bicoumaurins	Esculetin Psoralen Anthogenol Grandivittin Pseudocordatolide Isodispar B Dicoumarol	Seeds Roots Leaves Tonka bean	[42,43]
Tannins	Complex tannins Condensed tannins Ellagitannins Gallotannins	Tannic acid Chinese gallotannin Hexahydroxydiphenic acid	Bark Wood Leaves Fruit rRoots Plant galls Seeds	[44]
Phenolic acids	Hydroxycinnamic acids	Curcumin Caffeic acid Ferulic acid	Fruits Cereals	[45]
	Hydroxybenzoic acids	Gallic acid Protocatechuic acid Vanillic acid	Onion Raspberry Blackberry Strawberry	[45]
Flavonoids	Flavonols	Kaempferol Quercitin Myricetin	Onions Tea Lettuce Broccoli Apples	[46]
	Flavanones	Naringenin Hesperetin	Oranges Grapefruits	[47]
	Flavanols	Gallocatechin Catechins	Tea Red wine Chocolate	[48]
	Isoflavones	Genistein Glycitein Daidzein	Soybeans Legumes	[49]
	Anthocyanins	Pelargonidin Delphinidin Malvidin	Blackcurrant Strawberries Red wine Chokeberry	[50]
	Flavones	Apigenin Luteolin	Parsley Celery Red pepper Lemon Thyme	[51]
Stilbenes		Resveratrol	Red wine	[52]
Lignans		Pinoresinol	Flaxseed Sesame seed Red wine	[53]
		Lariciresinol Secoisolariciresinol Sesamin		

**Table 2:** In vitro modulation of gut microbiota through polyphenol consumption.

Polyphenol Source	Strains (spp)	Conditions	Method	Time (Fermentation/Incubation/Exposure)	Materials	Main Metabolites	Outcome	Ref.
Raspberry	N.S. ^1^	In vitro gastrointestinal digestion with heat-stable α-amylase at 25 °C, 30 min protease, at 95 °C, 35 min and with α-amyloglucosidase, 60 °C, 35 min)	In vitro fermentation	48 h fermentation	Fecal samples (healthy volunteers)	Propionic acid, butyric acid, acetic acid, isobutyric acid, isovaleric acid, valeric acid, isocaproic acid, caproic acid, and heptanoic acid	Polyphenols had a better prebiotic-like effect, in comparison with the fiber fractions ↑ ^3^ Bifodobacteria	[137]
Olive pomace	*Firmicutes*, *Lactobacillus spp*., *Enterococcus* spp., *Clostridium leptum*, *Bacteroidetes*, *Bacteroides* spp., *Prevotella* spp., *Bifidobacterium* spp.	In vitro simulations of gastrointestinal digestion A portion of the non-absorbable sample was lyophilized→ ^2^ exposed to fecal fermentation (fresh fecal inoculum)	In vitro simulated gastrointestinal digestion In vitro fecal fermentation	Samples were collected after 0, 12, 24, and 48 h of incubation	Feces (healthy volunteers)	Acetate, propionate, and butyrate	↑ SCFAs, potential antioxidant, and antimicrobial activity. Beneficial modifications were observed in *Firmicutes* and *Bacteroidetes* groups (after intervention)	[153]
Red and white grapes	*Lactobacillus*, *Bifidobacterium* for pure cultures; *B. longum*, *L. reuteri*, *B. vulgatus*, *Clostridium perfringens*, *Enterobacter cloacae* for mixed cultures	In vitro GI digestion (Infogest protocol) In vitro colonic fermentation assays The DNA extraction-with 1 mL of the sample using Realpure Microspin Real kit	In vitro GI digestion	48 h fermentation	Feces (healthy volunteers)	N.S.	↑ *Lactobacillus* and *Bifidobacterium* White grape polyphenolic extracts → ↑ for total bacteria and *Bifidobacterium* spp. Red grape polyphenolic extracts which showed significant changes for all the analyzed bacterial groups, without *Bacteroides* spp. ↑ *Firmicutes* and *Proteobacteria* from 0 to 48 h, both substrates	[138]
Predigested mango peel	*Bifidobacterium*, *Lactobacillus*, *Dorea*, *Lactococcus*	In vitro model of the colon (TIM-2) using human fecal microbiota and sampled after 0, 24, 48, and 72 h A carbohydrate mixture of Standard Ileal Effluent Medium (SIEM)—control	Dynamic in vitro model of the human colon	72 h experimental period	Fecal samples (healthy donors)	Acetic acid, propionic acid, butyric acid, valeric acid, formic acid, iso-valeric acid, ammonia	Mango peel fermentation → 80 bacterial genera identified ↑ *Bifidobacterium* with a maximum at 24 h fermentation; at 72 h mango peel favored ↑ *Bifidobacterium* and *Lactobacillus*	[141]
Green tea, oolong tea, and black tea	*Bifidobacterium*, *Lactobacillus/Enterococcus*, *Bacteroides-Prevotella*, *Clostridium histolyticum*	To obtain the fecal slurries it was necessary to mix fresh fecal + autoclaved phosphate buffered saline to yield 10% suspensions Green tea polyphenols, oolong tea polyphenols, black tea polyphenols, and fructooligosaccharides as the control group Fermentation—150 μL of fecal slurry to 1350 μL of culture medium	In vitro fermentation Intestinal absorption	72 h	Fecal samples (healthy volunteers)	Formic acid, acetic acid, propionic acid, butyric acid	↑ *Bifidobacterium* spp., oolong tea, and black tea had better effects than green tea Proliferation of *Lactobacillus/Enterococcus* spp. ↓ ^4^ *Firmicutes/Bacteroidetes* ratio and *Clostridium histolyticum*	[144]
Grape pomace (GP)	*Bifidobacteria*, *Lactobacillus*	Simulation of the effect of digestive tract was performed by dissolving 900 mg of the lyophilized GP extract into 20 mL of ultra-pure water In vitro fermentation was assessed using only 2 of the previous strains Samples of the fermentation broth were prelevated at 0, 4, 8, 24, and 48 h for metabolites’ analysis	In vitro stimulated gastrointestinal digestion	48 h fermentation	Syrah grape pomace	Acetic acid, butyric acid, formic acid, propionic acid	Until GI digestion, grape pomace extract proved to have antimicrobial activity against pathogenic bacteria	[140]
Pomegranate juice, pomegranate pulp, pomegranate peel extract	N.S.	In vitro digestion procedure applied The method consisted of a continuous-flow dialysis system performed with a dialysis tube	In vitro GI digestion In vitro fermentation	0, 2, 8, 24, 48, 72 h	Fresh fecal samples (three healthy adults)	Urolithin A, urolithin B, gallic acid, catechol, protocatechuic acid, coumaric acid	Pomegranate peel extract→ the best source of microbial substrates at the colonic level The use of pomegranate peel extracts obtained as a sub-product of the pomegranate juice industry → strategy to enrich or fortify (pomegranate products, fruit-based products) → enhancement of the pomegranate`s therapeutic effect (subjects with a low capacity to produce urolithin)	[148]
Pineapple	*Bifidobacteria*, *Lactobacillus*, *E. coli*, *Adlercreutzia equolifaciens*, *Asaccharobacter celatus*, *Slackia equolifaciens*, *Eubacterium limosum*, *Enterobacter*, *Escherichia*	In vitro digestion They were hydrolyzed with pepsin → gastric fraction Intestinal digestion (simulated by hydrolysis with pancreatin and α-amylase) The samples were centrifuged → the supernatants were brought to a volume of 50 mL → dialysis	In vitro gastrointestinal digestion Colonic fermentation	Samples were incubated and collected at 0, 6, 12, 24, and 48 h	Fecal samples (3 healthy adults)	Propionic acid, acetic acid, p-hydroxybenzoic acid, 3-hydroxybenzoic acid, 4-hydroxyphenyl acetic acid, p-hydroxybenzoic acid	The consumption of pineapple snack bars → the regulation of the antioxidant and anti-inflammatory effects - the presence of 4-hydroxyphenyl acetic acid ↓ anxiety and depression - p-hydroxybenzoic acid → potential therapeutic compound (could potentiate the anticancer role of adriamycin-breast cancer)	[151]
Red fruit extracts	*L. rhamnosus*, *L. paracasei*, *L. splantarum*, *Bacillus cereus*, *S. aureus*, *E. coli*, *Listeria monocytogenes*	Potential mechanisms involved in the inhibition of pathogenic bacterial growth analyzed with a well diffusion assay The kinetics growth was performed by using a modified de Man, Rogosa, Sharpe broth fermentation with red fruit extracts	In vitro fermentation	Growth conditions between 24–48 h	Collected from culture collection, human intestinal tract, isolated from food, probiotic strains combination	N.S.	↓ *B. cereus*, *S. aureus*, *E. coli* Almost all probiotics ↑ in the presence of red fruits extracts, except *L. paracasei* ↑ antioxidant potential of the probiotic-fruit extract combination	[154]
Pomegranate extract (POMx), pomegranate juice (POM juice)	*Bifidobacterium, Lactobacillus*, *Enterobacteriaceae*, *Bacteroides gragilis* group, clostridia, bifidobacteria, and lactobacilli	Aliquots of 10 μL of the homogenized stool specimens were inoculated into seven different test broths The test tubes were inoculated at 37° C for 6 days	In vitro culture tubes	Between 24 h and 7 days	Stool specimens from 8 healthy volunteers	Urolithins A and B, punicalagin A and B, punicalin, glycosyl ellagic acid	↑ *Bifidobacterium* and *Lactobacillus* (POMx) ↓ *B. fragilis* group, clostridia, and *Enterobacteriaceae*	[124]

^1^ N.S.—not specified, ^2^ →—next step, ^3^ ↑—increase, ^4^ ↓—decrease.

**Table 3:** In vivo modulation of gut microbiota through polyphenol consumption.

Polyphenol Source	Strains (spp)	Conditions	Method	Time (Fermenation/Incubation/Exposure)	Materials	Main Metabolites	Outcome	Ref.
Blueberry	*Proteobacteria*, *Deferribacteres*, *Actinobacteria*, *Bifidobacterium*, *Desulfovibrio*, *Adlercreutzia*, *Helicobacter*, *Flexispira*, *Prevotella*	Four groups: group A, a normal-fat diet, group B, a high-fat diet, group C, a high-fat diet supplement with polyphenol extract, and group D a high-fat diet supplemented with Orlistat, as a positive control The fecal DNA extraction using a DNA isolation kit	Administrated as a supplement (200 mg/kg body weight/day)	12 weeks	C57BL/6 J mice of 4 weeks	N.S. ^1^	Supplementation with polyphenol extract ↓ ^2^ the body weight of the high fat diet-fed mice by 6–7% ↑ ^3^ *Bifidobacterium*, *Desulfovibrio*, *Adlercreutzia*, *Helicobacter,* and *Flexispira*	[154]
Lyophilized jabuticaba seed extract (LJE)	*Firmicutes* *Bacteroidetes* *Proteobacteria*	Animals were treated to develop cancer (by administrating dimethylhydrazine dihydrochloride (DMH)) The non-induced animals received similar s.c. injections of EDTA solution The treatments: 10 mL/kg body weight, orally, by gavage	In vivo, experimental design	2 weeks	Wistar rats	Castalagin Vescalagin Procyanidin A Ellagic acid	↑ Bacteroidetes, ↓ Firmicutes (when DMH treated mice received the yogurt or the yogurt with LJE)	[162]
Grape extract	*Lachnoclostridium* *Blautia* *Bacteroides* *Lactobacillus* *Vibrio*	Divided in five groups and the samples were administered intragastrically three times/week The feces were collected at the moment 0 (before the treatment) and 28 days The microbiota comparison was determined using the analysis of the DNA of the fecal samples of the animal	Intragastrically administration	4 weeks	5 female BALB/c mice (5 weeks old)	N.S. ^1^	The microbiota was not affected by the sample composition or time of treatment No significant differences in bacterial composition and relative abundance	[157]
Tart cherries	*Verrucomicrobia*, *Synergistes*, *Akkermansia*, *Cloacibacillus*, *Bifidobacterium*, *Bilophila*, Firmicutes, Proteobacteria, *Collinsella*, *Assacharobacter*, *Bacteroides*, *Parabacteroides*	Participants consumed 237 mL of juice daily for 5 days Collection of stool sample before and a stool sample after the dietary intervention	In vivo human dietary intervention In vitro, fermentation	5 days	10 healthy participants (5 = male, 5 = female)	4-hydroxyphenylpropionic acids, 4-hydroxyphenylacetic acid, quercetin-3-O-glucoride, quercetin-3-O-rutinoside, kaempferol-3-O-rutinoside, 3,4 and 4-hydroxybenzoic acid	Gut microbiota strongly influences polyphenol metabolites Polyphenols in tart cherries and concentrates were to a certain extent metabolized by *Bifidobacterium*	[156]
Herbal tea: ginseng (GS), red ginseng (RGS), notoginseng (NGS), *Gynostemma pentaphyllum* (jiaogulan- GpS)	*Bacteroides*, *Lactobacillus*, *Bifidobacterium*, *Firmicutes*, *F. prasnitzii*, *Bacteroides*	Eight-week old male mice, 5 experimental groups, daily single dose of herbal saponins at 500 mg/kg or Milli-Q H_2_O by gavage for 15 consecutive days Feces collected, 8:00 to 10:00 a.m. on day 0, day 5, day 10, and day 15	In vivo-daily intake of herbal saponins	15 days	50 C5777BL/6 8 weeks old male mice	Butyrate	Ingested herbal saponins can increase the beneficial bacteria in the gut of the host ↓ *Firmicutes* on the GpS treatment group, ↑ *Bacteroidetes* the GpS and NGS group GpS, NGS, and GS ↑ *Lactobacillus*, whereas NGS and RGS ↑ *Bifidobacterium* ↑ *F*. *prausnitzii* in the GpS group	[149]
Red raspberry (polyphenolic extracts from whole fruit, seed, and pulp)	*Ruminococcus*, *Mogibacteriaceae*, *Bifidobacterium*, *Coriobacteriaceae*, *Verrucomicrobia*, *Bacteroidetes*, *Actinobacteria*, *Proteobacteria*, *Akkermansia*, *Clostridiales*, *Dehdobacterium*, *Lachnospiraceae*, *Roseburia*, *Adlercreutzia*	Five groups: a low-fat diet, high-fat diet, high-fat diet supplemented with 0.4% by weight-red raspberry (RR) whole fruit polyphenols, 0.1% by weight RR seed polyphenols, 0.3% by weight RR seed polyphenols Mice were fed for 16 weeks ad libitum	Administration of different types of diets	16 weeks	C57BL/6 male mice	Butyrate, pentahydroxy-urolithin, tetrahydroxy-urolithin	High-fat diets with RR polyphenols have a prebiotic effect on the gut microbiota	[155]
Red wine polyphenols	*Bifidobacterium*, *Enterococcus*, *Eggerthella lenta*	Fecal, and 24 h urine samples (at baseline and after each intervention period) metabolites in urine were analyzed by UPLC-MS/MS Extraction of DNA was from 200 mg stools by using a QIAmp DNA Stool Mini Kit	Consumption of red wine, dealcoholized red wine, and gin	Three consecutive periods of 20 days each with an initial washout period	9 adult men	Syringic acid, p-coumaric acid, 4-hydroxybenzoic acid, homovanillic acid, hydroxycinnamates, 3,4-dihydroxyphenylacetic acid	Bacterial changes after red wine consumption (±alcohol) have been associated with the excretion of phenolic metabolites Phenolic compounds are important in the maintenance of intestinal health	[158]
Red wine polyphenols	*Bifidobacterium*, *Lactobacillus*, *F. prausnitzii*, *Roseburia*, *Escherichia coli*, *Enterobacter cloacae*	Four periods: the participants were given a two-week washout period during which they did not consume any red wine, followed by two 30-day intervention periods during which they drank just red wine (272 mL/day) or dealcoholized red wine (272 mL/day), separated by a 5-day washout phase Three different fecal samples were provided by each participant, at baseline, after the washout period, and at the end DNA extraction from 200 mg of stools (performed with QIAamp DNA stool Mini Kit)	In vivo study (intake of red wine (RW) and dealcoholized red wine (DRW) polyphenols)	Two weeks washout period, 2 periods of 30 days each, and between a period of 15 days	Twenty adults (10 met the criteria for metabolic syndrome (MetS), and 10 healthy)	N.S.	↑ *Blautia coccoides*, *F*. *prausnitzii*, *Roseburia*, and *Lactobacillus*, *↓ Clostridium histolyticum* After RW and DRW intake ↓ Bacteroides, ↑ *Prevotella*, *Bifidobacterium*, and *Eggerthella lenta* ↓ *Echerichia coli* and *Enterobacter cloacae* in MetS group	[159]
*Cyclorarya paliurus* flavonoids	*Prevotellaceae*, *Bacteroidaceae*, *Ruminococcaceae*, *Lachnospiraceae*, *Veillonellaceae*, *Enterobacteriaceae*	To obtain a human intestinal microbial suspension, the supernatants prepared from each volunteer’s fecal sample were combined Three groups: control group (CONT group), the constant darkness group (CD group), and the constant darkness with flavonoid supplementation group (CPF group) Fecal samples were collected at baseline and 4 weeks after they were divided	In vivo, administration by gavage	Four weeks	Germ-free 6-week-old C57BL/6J male mice; 6 healthy volunteers	-	The diversity of the total bacterial community ↑ during CPF treatment ↓ Firmicutes/Bacteroidetes ratio in CPF treatment After 4 weeks CPF group: ↑*Prevotellaceae*, and *Bacteroidaceae*, and ↓ *Ruminococcaceae*, *Lachnospiraceae*, and *Veillonellaceae* In the CPF group ↑ *Prevotella*, and *Bacteroides*, and ↓ *Faecalibacterium*, *Mitsuokella*, *Ruminococcus*, *Desulfovibrio*, *Megamonas*	[166]
Carrot	*Firmicutes*, *Bacteroidetes*, *Proteobacteria*	Three groups: control group (CON), carrot dietary fiber (CDF), dephenolized carrot dietary fiber (CDF-DF) The CDF and CDF-DF groups, with daily intake of approximately 0.6 g in 200 μL of CDF and CDF-DF, by oral administration for 7 consecutive days Fecal slurries: homogenizing the fecal samples with pH 7.0, 0.1 M sodium phosphate buffer followed by filtration	In vivo, oral administration	Seven days	Male BALB/c mice; 3 healthy donors	Acetic acid, butyric acid, propionic acid, valeric acid	CDF-fed mice: ↑*Bacteroides*, and ↓ *Proteobacteria* The CDF group: ↓ *Clostridiales*, *Coprococcus*, *Oscillospira*, and *Dehalobacterium*, *↑ Lactobacillus* compared to those in the CDF-DF, and CON groups	[168]

^1^ N.S.—not specified, ^2^ ↓—decrease, ^3^ ↑—increase.
